# ADAMTS2 drives prostate cancer progression by activating FAK/PI3K/AKT signaling and suppressing ferroptosis via COL1A1

**DOI:** 10.3389/fonc.2026.1784882

**Published:** 2026-04-27

**Authors:** Jinzhuo Ning, Jinrun Wang, Lizhe Xu, Zhiyan Zhou, Haoyong Li

**Affiliations:** 1 Department of Urology, Renmin Hospital of Wuhan University, Wuhan, Hubei, China; 2 Department of Urology, The First Affiliated Hospital of Hainan Medical University, Haikou, Hainan, China

**Keywords:** ADAMTS2, COL1A1, FAK/PI3K/AKT signaling, ferroptosis, prostate cancer

## Abstract

**Background:**

ADAMTS2, a secreted metalloproteinase essential for collagen maturation, exhibits context-dependent roles in cancer but remains uncharacterized in prostate cancer (PCa). Its potential involvement in PCa progression and underlying mechanisms are unknown.

**Methods:**

We integrated bioinformatic analysis of TCGA-PRAD data with clinical specimen validation, *in vitro* functional assays (proliferation, migration, invasion), co-immunoprecipitation (Co-IP), ferroptosis sensitivity testing, pharmacological inhibition, and *in vivo* xenograft models to investigate ADAMTS2’s expression, function, and molecular mechanisms in PCa.

**Results:**

ADAMTS2 was significantly upregulated in PCa tissues and cell lines, correlating with aggressive clinicopathological features and poor progression-free survival. Functionally, ADAMTS2 promoted PCa cell aggressiveness *in vitro* and tumor growth *in vivo*. Mechanistically, ADAMTS2 directly interacted with and upregulated COL1A1, leading to stimulation of the FAK/PI3K/AKT pathway. This cascade, in turn, enhanced the expression of ferroptosis defense proteins (SLC7A11 and GPX4), suppressed lipid peroxidation, and conferred resistance to ferroptosis. Pharmacological inhibition of FAK reversed both the oncogenic and anti-ferroptotic effects of ADAMTS2.

**Conclusions:**

Our investigation considers ADAMTS2 as a novel oncogenic driver in PCa that promotes tumor progression by reinforcing a tumor-permissive extracellular matrix through upregulation of COL1A1 and by stimulating the FAK/PI3K/AKT pathway to suppress ferroptosis. These findings position ADAMTS2 as a potential predictive biomarker and a valuable therapeutic target for defeating ferroptosis resistance in PCa.

## Introduction

Prostate cancer (PCa) is the most diagnosed malignancy among men in many developed countries and a main cause of cancer-correlated death globally (1–4). It arises from the epithelial cells of the prostate gland and typically exhibits a heterogeneous clinical course, ranging from indolent, slow-growing tumors to aggressive, metastatic disorder (5). While early-stage PCa is often asymptomatic and detectable through raised prostate-specific antigen (PSA) levels or digital rectal test, advanced disorder can cause significant morbidity, including bone metastases and urinary obstruction (6). Despite advances in screening and treatment, current diagnostic and prognostic tools remain imperfect, underlining the crucial requirement for elucidating the molecular and cellular mechanisms driving prostate carcinogenesis and progression.

The ADAMTS (A Disintegrin And Metalloproteinase with Thrombospondin Motifs) family comprises secreted zinc-dependent metalloproteinases that play critical roles in extracellular matrix (ECM) remodeling, organogenesis, and tissue homeostasis (7). Among its members, ADAMTS2 is best characterized as a procollagen N-proteinase responsible for the proteolytic processing of type I, II, and III procollagens, thereby facilitating proper collagen fibril assembly (8). Beyond its canonical role in connective tissue biology, emerging evidence implicates ADAMTS2 in tumorigenesis: it acts as an oncogenic driver in ovarian and gastric cancers, where its upregulation is associated with poor prognosis and enhanced tumor invasiveness (9, 10). In contrast, its expression and functional relevance in PCa remain entirely unexplored. Given the crucial function of ECM dynamics and stromal interactions in PCa progression, the potential involvement of ADAMTS2 warrants systematic investigation (11).

The FAK/PI3K/AKT signaling pathway is a canonical cascade governing fundamental cellular processes, including proliferation, survival, and metastasis in various malignancies (12). Emerging evidence indicates that ADAMTS family members are intricately linked to this signaling axis. For instance, ADAMTS-1 has been reported to modulate the PI3K/Akt-eNOS-VEGF pathway in lung cancer (13), while ADAMTS-12 functions as a key driver of bladder cancer progression by activating FAK/PI3K/AKT signaling (14). Additionally, studies have shown that ADAMTS-8 expression is induced by IL-6 via the PI3K pathway (15). Notably, our group recently demonstrated that COL8A1 promotes PCa progression by activating the FAK/PI3K/AKT pathway specifically through the regulation of ADAMTS2 (16). Based on these antecedents, we hypothesized that ADAMTS2 serves as a critical upstream regulator of the FAK/PI3K/AKT pathway to drive PCa aggressiveness.

In this study, we aim to determine whether ADAMTS2 drives PCa progression through a molecular axis involving its interaction with COL1A1, subsequent stimulation of the FAK/PI3K/AKT pathway, and consequent inhibition of ferroptosis. By combining clinical data analysis, *in vitro* functional assays, protein interaction studies, pathway inhibition, and *in vivo* xenograft models, we seek to establish ADAMTS2 as a key regulator linking extracellular matrix modulation to ferroptosis resistance in PCa.

## Materials and methods

### Bioinformatic analysis

To explore the expression and clinical relevance of ADAMTS2 in PCa, we assessed RNA-seq data from The Cancer Genome Atlas Prostate Adenocarcinoma (TCGA-PRAD) cohort. Specifically, the TCGA-PRAD dataset comprised a total of 553 RNA-seq samples, which included 501 tumor tissues and 52 adjacent normal tissues. Clinical data were available for 500 patients, and all 553 RNA-seq samples had corresponding clinical information mapping, noting that 4 RNA-seq samples were derived from the same patients. Furthermore, to validate the expression pattern of ADAMTS2 in an independent cohort, the GSE3325 dataset was obtained from the Gene Expression Omnibus (GEO) database. We then compared ADAMTS2 expression between tumor and neighboring adjacent non tumoral tissues across these cohorts. Correlations between ADAMTS2 expression and clinicopathological features—such as T and N stages, Gleason score, residual tumor status, and patient age—were assessed using appropriate statistical tests. Kaplan–Meier survival analysis was conducted to assess the link between ADAMTS2 levels and progression-free interval (PFI). Differentially expressed genes co-expressed with ADAMTS2 were identified using Spearman correlation analysis, and functional enrichment was conducted via Gene Ontology (GO) and Kyoto Encyclopedia of Genes and Genomes (KEGG) pathway analyses. To explore potential biological pathways related to ADAMTS2 expression, particularly those related to PI3K/AKT signaling and ferroptosis, Gene Set Enrichment Analysis (GSEA) was utilized. Protein–protein interaction (PPI) networks were created via the STRING to predict functional associations between ADAMTS2 and candidate partners such as COL1A1.

### Clinical samples

Paired PCa tumor tissues and adjacent non tumoral tissues (n = 6) were acquired from patients who underwent radical prostatectomy at Renmin Hospital of Wuhan University. The inclusion criteria were as follows: (1) a primary diagnosis of prostate adenocarcinoma confirmed by pathological examination; (2) no history of preoperative chemotherapy, radiotherapy, or androgen deprivation therapy; and (3) sufficient tissue available for both molecular analysis and pathological verification. The patient cohort had a median age of 68 years (range 61–76 years). The clinicopathological characteristics of the patients included clinical stages T2b to T3a and Gleason scores of 7 (3 + 4 or 4 + 3) to 9. For tissue segregation, all specimens were meticulously evaluated and partitioned by experienced senior pathologists at the Department of Pathology. Tumor tissues were harvested from the core of the neoplastic lesion, while adjacent non tumoral tissues were obtained from the macroscopic normal area at least 2 cm away from the tumor margin. The identity and purity of each tissue sample were confirmed through routine clinical histopathological examination (H&E staining) by the hospital’s pathology department before the samples were snap-frozen in liquid nitrogen and stored at −80 °C. The Institutional Ethics Committee of Renmin Hospital of Wuhan University approved all procedures (approval no: WDRY2021-KS010), and informed consent was obtained from all participants.

### Cell culture

The human normal prostate epithelial cell line RWPE-1 was cultivated in Keratinocyte Serum-Free Medium (K-SFM; Gibco, USA, 10744019) enriched with 0.05 mg/mL bovine pituitary extract and 5 ng/mL epidermal growth factor, following the manufacturer’s instructions. PCa cell lines PC-3, 22Rv1, and DU145 were kept in RPMI-1640 medium (Gibco, USA, 11875093) with 10% fetal bovine serum (FBS; Gibco, USA, A5669401) and 1% penicillin–streptomycin (Gibco, USA, 15140148). An incubation of cells was conducted at 37 °C in a humidified 5% CO_2_ environment and routinely tested for mycoplasma contamination.

### Cell transfection and lentiviral infection

Stable overexpression or knockdown of ADAMTS2 and COL1A1 in PCa cells was achieved using lentiviral vectors. The lentiviral particles carrying ADAMTS2 or COL1A1 overexpression constructs, as well as short hairpin RNAs (shRNAs) targeting ADAMTS2 or COL1A1, were acquired from GeneChem (China). For viral infection, PCa cells were seeded into 6-well plates at a density of 2×10^5^ cells per well and cultured until reaching appropriate confluence. We utilized commercial viral particles (packaged in HEK293T cells by the manufacturer) with a titer of 1×10^9^ TU/mL. Infection was performed once at a multiplicity of infection (MOI) of 20 in the presence of 8 μg/mL polybrene (GeneChem). After 24 h, the medium was substituted with fresh medium. Based on the manufacturer’s recommended protocols and our preliminary observation of selection efficiency, stable cell lines were selected with 4 μg/mL puromycin for 5 days, a concentration confirmed to effectively eliminate non-infected cells in these specific lines.

### Chemicals

The ferroptosis inducers erastin (Cat. No. HY-15763) and RSL3 (Cat. No. HY-100218A), as well as the FAK inhibitor Y15, were all acquired from MedChemExpress (Shanghai, China). The preparation of stock solutions was conducted in dimethyl sulfoxide (DMSO) and then storing at −80 °C was conducted. Working concentrations were freshly diluted in complete culture medium immediately before use, with the final DMSO level not exceeding 0.1%.

### Western blot analysis

For *in vitro* experiments, cells were seeded into 6-well plates at a density of 3×10^5^ cells per well and cultured for 48 h before total protein isolation was conducted from the cultured cells or frozen tumor tissues via RIPA lysis buffer enriched with protease and phosphatase inhibitors (Beyotime, China, P0013B). Protein levels were detected via a BCA Protein Assay Kit (Beyotime, China, P0012). The separation of equal quantities of protein (20 μg) was conducted by SDS-PAGE, followed by transferring onto PVDF membranes (Millipore, USA). After a 1-h blockage with 5% non-fat milk in TBST at room temperature, membranes were introduced into an incubator at 4 °C overnight with primary antibodies: ADAMTS2 (1:5000, Ag34957, Proteintech, USA), TPX2 (1:2000, 11741-1-AP), FAK (1:20000, 12636-1-AP), phospho-FAK (p-FAK, 1:1000, 83933-1-RR) (All from Proteintech, USA), PI3K (1:2000, ab302958), phospho-PI3K (p-PI3K, 1:2000, ab191606), AKT (1:2000, ab8933), phospho-AKT (p-AKT, 1:2000, ab38449), SLC7A11 (1:2000, ab307601), GPX4 (1:2000, ab125066), and GAPDH (1:2000, ab9485) (All from Abcam, UK). After washing, a 1-h incubation of membranes was conducted with HRP-conjugated secondary antibodies at room temperature. Protein bands were observed via an ECL chemiluminescent detection system (Bio-Rad, USA) and ImageJ (NIH, USA) was utilized for quantification. GAPDH was considered the loading control.

### Quantitative real-time polymerase chain reaction

Total RNA isolation was conducted from cells or tissues via TRIzol reagent (Invitrogen, USA, 15596026CN) following the manufacturer’s protocol. RNA level and purity were determined with a NanoDrop spectrophotometer (Thermo Fisher Scientific, USA). Complementary DNA (cDNA) synthesis was conducted from total RNA (1 μg) via the PrimeScript™ RT Master Mix (Takara, Japan). A QuantStudio™ 5 Real-Time PCR System (Applied Biosystems, USA) with SYBR Green PCR Master Mix (Takara) was utilized to conduct qRT-PCR. The synthesis of primers for ADAMTS2, COL1A1, and other target genes was conducted by Sangon Biotech (China), and **Table 1** lists them. GAPDH acted as the internal control, and relative levels were assessed via the 2^^-^ΔΔCt^ technique.

**Table 1:** Primer sequences for qRT-PCR.

Gene	Forward (5’-3’)	Reverse (5’-3’)
ADAMTS2	GCCTAGCCGAAGCCTCCTC	GAAGAACTCCTCCTCCTCCATCC
COL1A1	CCTGGTGATGCTGGTGCTAA	ACATTACCAATGGGGCCAGG
GAPDH	GTGGACCTGACCTGCCGTCTAG	GAGTGGGTGTCGCTGTTGAAGTC

### Cell viability assay

Cell viability assessment was conducted via the CCK-8 assay (Beyotime, China, C0037). Cells were seeded into 96-well plates at a density of 2000 cells per well in 100 μL of complete medium. Cell viability was assessed at 24, 48, 72, and 96 h post-seeding. At each time point, the CCK-8 reagent was applied (10 μL/well) and a 1-h incubation was conducted at 37 °C. Subsequently, absorbance was read at 450 nm via a microplate reader.

### Wound healing assay

Cells were seeded into 6-well plates at a density of 4×10^5^ cells per well and cultivated until approaching 90–100% confluence. A sterile 200 μL pipette tip was utilized to form a straight scratch wound across the monolayer. The cells were then rinsed twice with PBS to eliminate detached debris and replenished with new serum-free medium to arrest cell proliferation and eliminate bias from cell division during the migration period. An inverted phase-contrast microscope (Olympus, Japan) was utilized to capture photographs of wound area at 0 and 24 h. The quantification of wound closure rate was conducted by calculating the remaining wound area with ImageJ and normalized to the initial wound area at 0 h.

### Transwell assay

Cell migration and invasion were evaluated via 24-well Transwell chambers with 8-μm pores (Corning, USA). For migration assessments, 3 × 10^4^ cells in 200 μL serum-free medium were seeded in the top chamber. For invasion assessments, pre-coating of the chambers was conducted with Matrigel (BD Biosciences, USA) diluted 1:8 in serum-free medium, and an equal number of cells was applied. The bottom chamber comprised complete medium with 10% FBS as a chemoattractant. After incubation for 24 h at 37 °C, a cotton swab was utilized to eliminate the non-migrated or non-invaded cells. Cells that traversed to the bottom surface underwent fixation with 4% paraformaldehyde and staining with 0.1% crystal violet. Images of five randomly selected fields per insert were captured using an inverted phase-contrast microscope (Olympus, Japan). To ensure objective and rigorous quantification, the number of migrated or invaded cells was counted using the automated particle analysis function of ImageJ software (NIH, USA) with a consistent thresholding protocol, thereby eliminating manual counting bias.

### Co-IP analysis

Co-IP assessments were conducted to evaluate PPI between ADAMTS2 and COL1A1 under endogenous and exogenous conditions. For exogenous Co-IP, HEK293T cells were seeded into 10-cm dishes at a density of 2×10^6^ cells per dish. After 24 h, co-transfection was conducted with Flag-tagged ADAMTS2 and HA-tagged COL1A1 plasmids via Lipofectamine 3000 (Invitrogen, USA, L3000015). After 48 h, cell lysis was conducted in NP-40 buffer (50 mM Tris-HCl, pH 7.4; 150 mM NaCl; 1% NP-40; 1 mM EDTA) containing protease inhibitors. Lysates were pre-cleared with Protein A/G agarose beads (Santa Cruz Biotechnology) for 1 h at 4 °C, then an overnight incubation was conducted at 4 °C with anti-Flag or anti-HA antibodies. Immune complexes underwent capturing with Protein A/G beads for 2–4 h, washing four times, and eluting by boiling in 2× SDS loading buffer. For endogenous Co-IP, DU145 cell lysis was conducted under identical circumstances, and immunoprecipitation of lysates was conducted with anti-ADAMTS2 antibody or control IgG, followed by pull-down with Protein A/G beads.

### Measurement of malondialdehyde levels

MDA levels were assessed via a commercial MDA assay kit (Beyotime, China, S0131S) depending on the thiobarbituric acid (TBA) technique. Cells were seeded into 6-well plates at a density of 3×10^5^ cells per well and cultured for 48 h. Cell lysates underwent mixing with TBA reagent, heating at 95 °C for 60 min, and ice-cooling. Absorbance was read at 532 nm, and MDA level was evaluated from a standard curve and normalized to total protein.

### Measurement of glutathione levels

Intracellular reduced glutathione (GSH) and oxidized glutathione (GSSG) levels were assessed via a GSH/GSSG assay kit (Beyotime, China, S0053) as per the manufacturer’s guidelines. Cells were seeded into 6-well plates at a density of 3×10^5^ cells per well and treated as indicated for 48 h. Cell lysis was conducted in ice-cold assay buffer, and deproteinized supernatants were utilized for analysis. Total glutathione (GSH/GSSG) and GSSG were assessed spectrophotometrically at 412 nm, and GSH levels were measured by the subtraction of GSSG from total glutathione.

### Xenograft tumor formation assay

A subcutaneous injection of a mixture of 5 × 10^6^ DU145 cells in 100 μL of PBS with Matrigel (1:1) was introduced into the right flank of BALB/c nude mice (4–6 weeks old, male). Tumor growth was observed every seven days with caliper measurements. The sacrifice of mice was conducted 4 weeks post-implantation, and tumors were removed and weighed. The Institutional Animal Care and Use Committee approved all animal procedures carried out as per ethical protocols (Approval No. 20250101D).

### Statistical analysis

All bioinformatic statistical analyses were conducted using R software (version 4.2.1). For transcriptomic data, RNA-seq datasets (in TPM or FPKM formats) were processed with a log2(value+1) transformation. The comparison of ADAMTS2 expression between tumor and adjacent non tumoral tissues was evaluated using the Wilcoxon rank-sum test (via the stats and car packages). For survival analysis regarding the PFI, the optimal cut-off value for continuous gene expression was determined using the surv_cutpoint function in the survminer package, followed by Kaplan-Meier curve estimation and Cox proportional hazards regression via the survival package. Gene co-expression correlations were calculated using Spearman’s rank correlation coefficient, with p-values adjusted using the Benjamini-Hochberg (BH) method. Data visualization was primarily performed using the ggplot2 package. For *in vitro* and *in vivo* experimental data, results are presented as the mean ± SD from a minimum of three independent trials. Differences between two groups were evaluated using a two-tailed Student’s t-test, while comparisons among multiple groups were performed using one-way ANOVA followed by Tukey’s *post hoc* test. GraphPad Prism 9.0 was employed for the statistical analysis of experimental data. A p-value < 0.05 was considered statistically significant.

## Results

### ADAMTS2 is elevated in PCa tissues and cell lines

**Figure 1:**
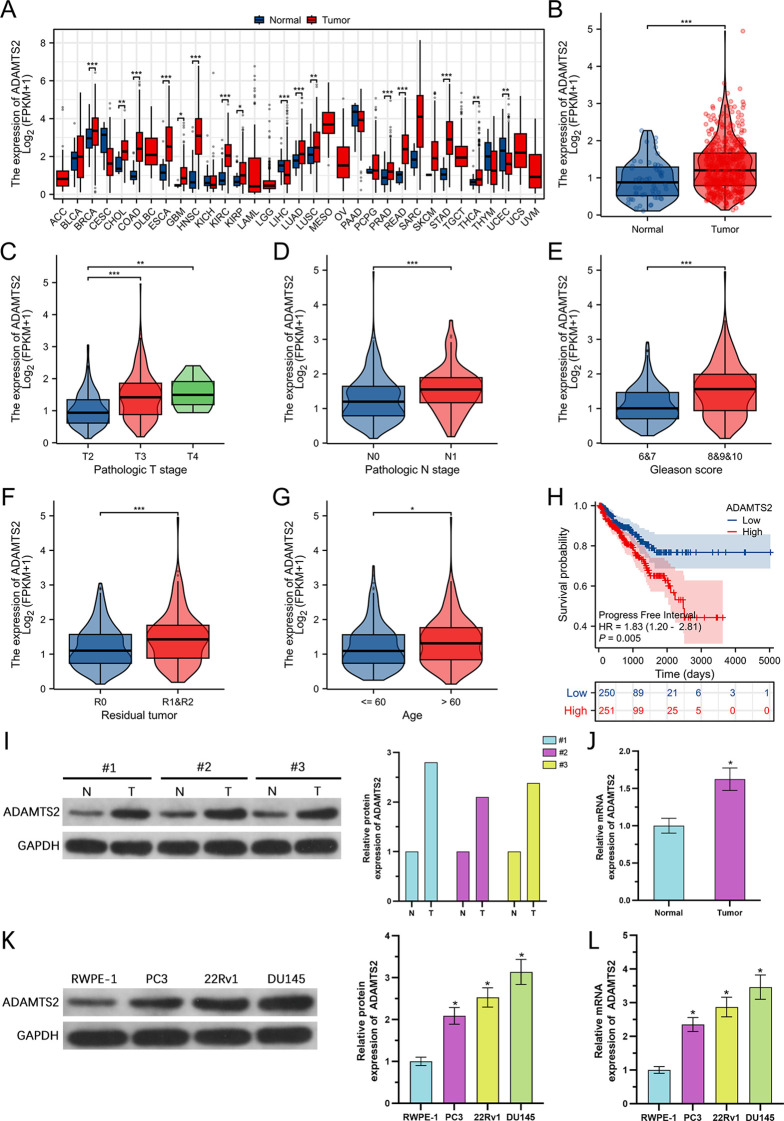
ADAMTS2 is overexpressed in PCa tissues and cell lines. **(A)** Pan-cancer analysis of ADAMTS2 mRNA expression levels across the TCGA dataset. **(B)** ADAMTS2 mRNA expression in PCa tissues compared to adjacent non-tumoral tissues from the TCGA-PRAD cohort. **(C–G)** Association of ADAMTS2 expression with clinicopathological features in PRAD, including clinical T stage, N stage, Gleason score, residual tumor status, and patient age. **(H)** Kaplan–Meier analysis of PFI based on high vs. low ADAMTS2 expression levels in PRAD patients. **(I, J)** Validation of ADAMTS2 expression in paired human PCa and adjacent non-tumoral tissues. mRNA levels were quantified by qRT-PCR (n=6 pairs), and protein levels were assessed by WB (*n*=3 pairs; representative bands are shown). **(K, L)** Comparison of ADAMTS2 mRNA and protein levels between a normal prostate epithelial cell line (RWPE-1) and three PCa cell lines (PC3, 22Rv1, and DU145). All cell-based quantitative data are presented as the mean ± SD from three independent biological replicates (n=3). Statistical significance was determined using one-way ANOVA followed by Tukey’s *post hoc* test. *P < 0.05, **P < 0.01, ***P < 0.001.

The TCGA pan-cancer dataset analysis showed that ADAMTS2 mRNA was significantly overexpressed across multiple tumor types compared to matching adjacent non tumoral tissues (**Figure 1A**). In the TCGA-PRAD cohort, ADAMTS2 expression was markedly higher in PCa tissues than in neighboring adjacent non tumoral tissues (**Figure 1B**). To validate this finding in an independent cohort, we analyzed the GSE3325 dataset, which consistently demonstrated significantly elevated ADAMTS2 levels in PCa tumor tissues compared to adjacent benign tissues (**Supplementary Figure 1A**). Clinicopathological correlation analysis illustrated that ADAMTS2 overexpression was significantly linked to advanced T and N stages, higher Gleason score, residual tumor presence, and older age (**Figures 1C–G**), indicating a potential role in PCa progression. Notably, when further stratifying patients with Gleason score 7 using the ISUP grading system, we found that ADAMTS2 expression was significantly higher in the 4 + 3 (ISUP Grade Group 3) subgroup than in the 3 + 4 (ISUP Grade Group 2) subgroup (**Supplementary Figure 1B**), further supporting its correlation with increased histological malignancy. Consistently, Kaplan–Meier survival analysis illustrated that individuals with elevated ADAMTS2 expression exhibited significantly shorter PFI than those with reduced expression (**Figure 1H**). To confirm these outcomes at the protein level, we performed WB and qRT-PCR on paired clinical specimens from PCa patients. Both assays verified that ADAMTS2 was significantly overexpressed in tumor tissues relative to matched neighboring adjacent non tumoral tissues (**Figures 1I, J**). Furthermore, assessment of ADAMTS2 expression in human prostate epithelial cells (RWPE-1) and PCa cell lines revealed markedly increased mRNA and protein levels in all cancer cell lines compared to RWPE-1, with the highest level detected in 22Rv1 and DU145 cells (**Figures 1K, L**). Based on these results, 22Rv1 and DU145 cells were chosen for subsequent functional experiments.

### ADAMTS2 overexpression promotes the aggressiveness of PCa cells

**Figure 2:**
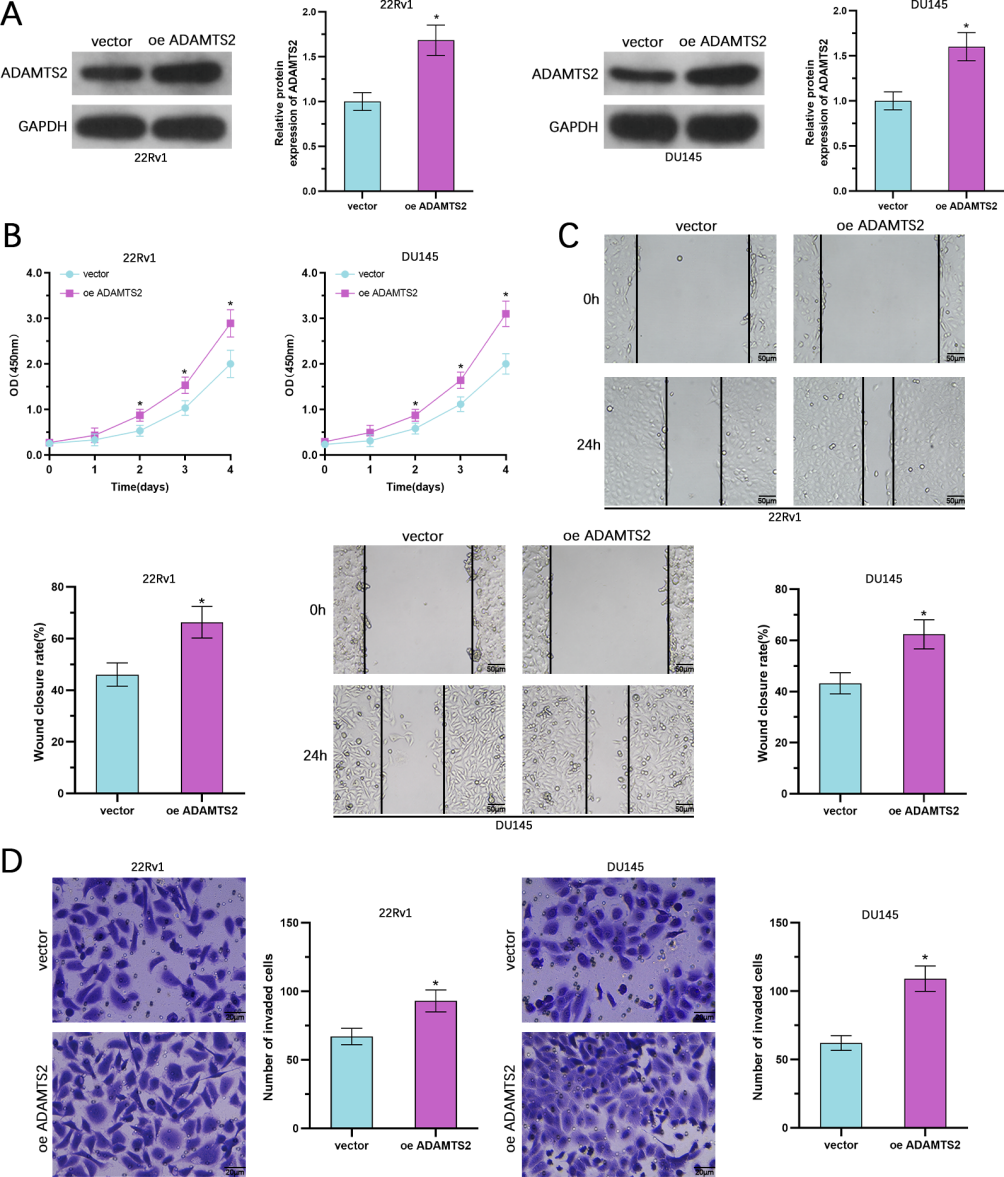
ADAMTS2 overexpression triggers the aggressiveness of PCa cells. **(A)** WB of ADAMTS2 overexpression in stable lentiviral-transduced 22Rv1 and DU145 cells. **(B)** Cell growth was estimated by CCK-8 assessment at 24, 48, 72, and 96 h post-seeding. The assay was performed under serum-free conditions to exclude the confounding effect of cell proliferation. (n=3; two-way ANOVA followed by Sidak’s multiple comparisons test). **(C)** Wound healing assay evaluating cell migration capacity. **(D)** Matrigel-coated Transwell assay measuring cell invasive potential. Data are shown as the mean ± SD from three independent biological replicates (n=3; two-tailed Student’s t-test). *P < 0.05.

To examine the ADAMTS2 function in PCa progression, we established stable ADAMTS2-overexpressing 22Rv1 and DU145 cell lines using lentiviral transduction. Additionally, given the relatively low endogenous expression of ADAMTS2 in PC3 cells, we also generated ADAMTS2-overexpressing PC3 cells to further validate the gain-of-function phenotype. WB verified efficient upregulation of ADAMTS2 in both cell lines (**Figure 2A**, **Supplementary Figure 1C**). Functional assessments illustrated that ADAMTS2 upregulation significantly enhanced cell growth, as evidenced by increased absorbance in CCK-8 assessments at 24, 48, 72 and 96 h post-seeding compared to control cells (**Figure 2B**, **Supplementary Figure 1D**). In wound healing assays, ADAMTS2-overexpressing cells exhibited accelerated wound closure, indicating enhanced migratory capacity (**Figure 2C**, **Supplementary Figure 1E**). Moreover, Matrigel-based Transwell invasion assays demonstrated that ADAMTS2 overexpression markedly increased the number of invading 22Rv1 and DU145 cells (**Figure 2D**), as well as PC3 cells (**Supplementary Figure 1F**). Collectively, these findings indicate that ADAMTS2 exerts oncogenic effects by promoting the aggressiveness of PCa cells *in vitro*.

### Suppression of ADAMTS2 suppresses the aggressiveness of PCa cells

**Figure 3:**
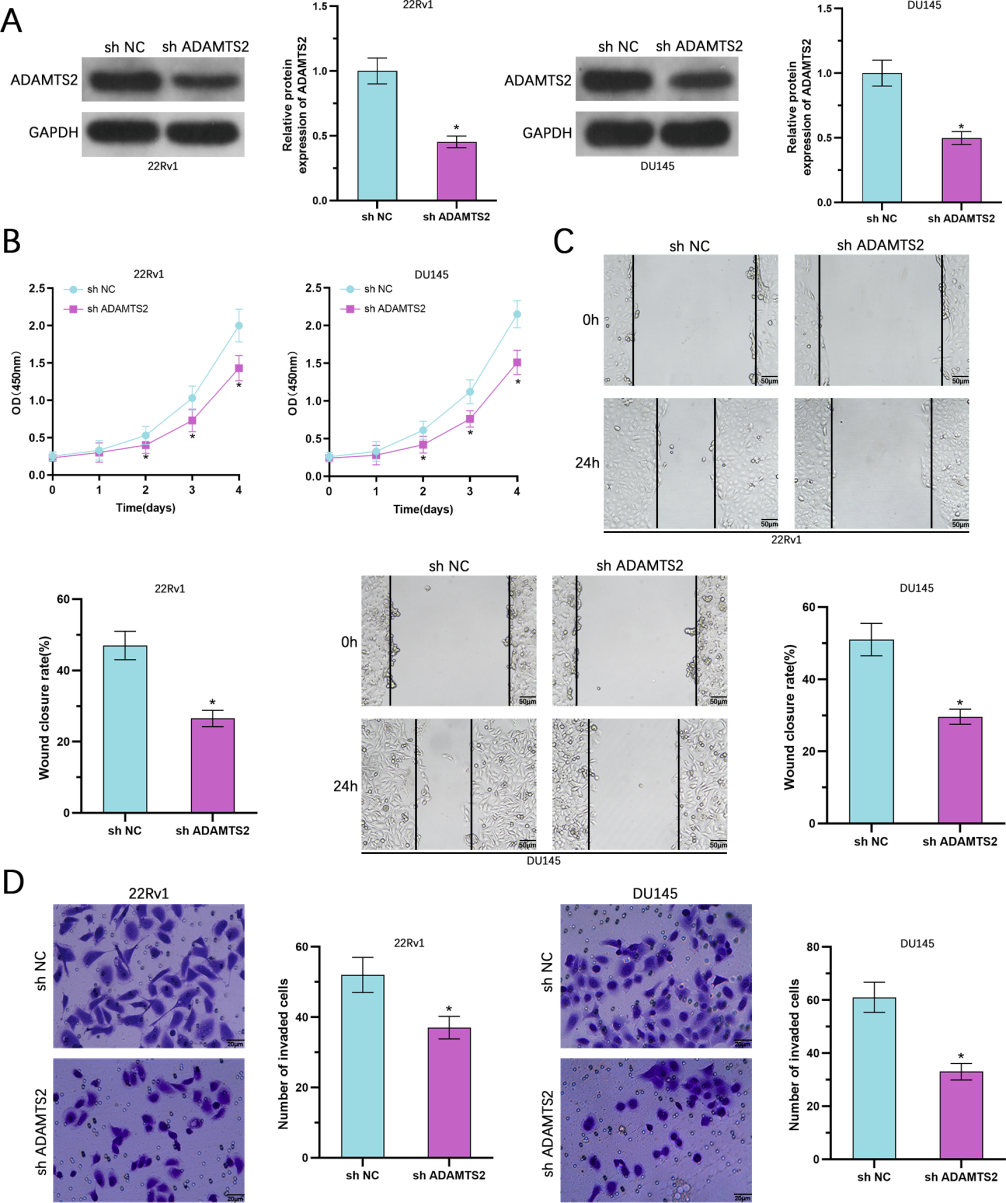
Suppression of ADAMTS2 suppresses the aggressiveness of PCa cells. **(A)** WB of ADAMTS2 knockdown in stable shRNA-expressing 22Rv1 and DU145 cells. **(B)** Cell growth was evaluated by CCK-8 assessment at multiple time points. **(C)** Wound healing assay evaluating cell migration after ADAMTS2 silencing. The assay was performed under serum-free conditions to exclude the confounding effect of cell proliferation. (n=3; two-way ANOVA followed by Sidak’s multiple comparisons test). **(D)** Matrigel-coated Transwell assay measuring invasive capacity upon ADAMTS2 knockdown. Data are shown as the mean ± SD from three independent biological replicates (n=3; two-tailed Student’s t-test). *P < 0.05.

To further corroborate the oncogenic function of ADAMTS2, we generated stable ADAMTS2-knockdown 22Rv1 and DU145 cell lines using lentiviral vectors expressing specific shRNAs. WB verified efficient silencing of ADAMTS2 protein expression in both cell lines (**Figure 3A**). Functional assays demonstrated that ADAMTS2 depletion significantly impaired cell proliferation, as shown by reduced CCK-8 absorbance over time compared to control cells (**Figure 3B**). Wound healing assays revealed a marked decrease in migration capacity upon ADAMTS2 knockdown (**Figure 3C**). Likewise, Transwell invasion assays indicated that silencing ADAMTS2 dramatically lowered the count of invading cells in both 22Rv1 and DU145 lines (**Figure 3D**). These results, in conjunction with the gain-of-function experiments, provide compelling evidence that ADAMTS2 is functionally required for the aggressive phenotypes of PCa cells.

### Determination and functional enrichment of ADAMTS2-coexpressed genes in PCa

**Figure 4:**
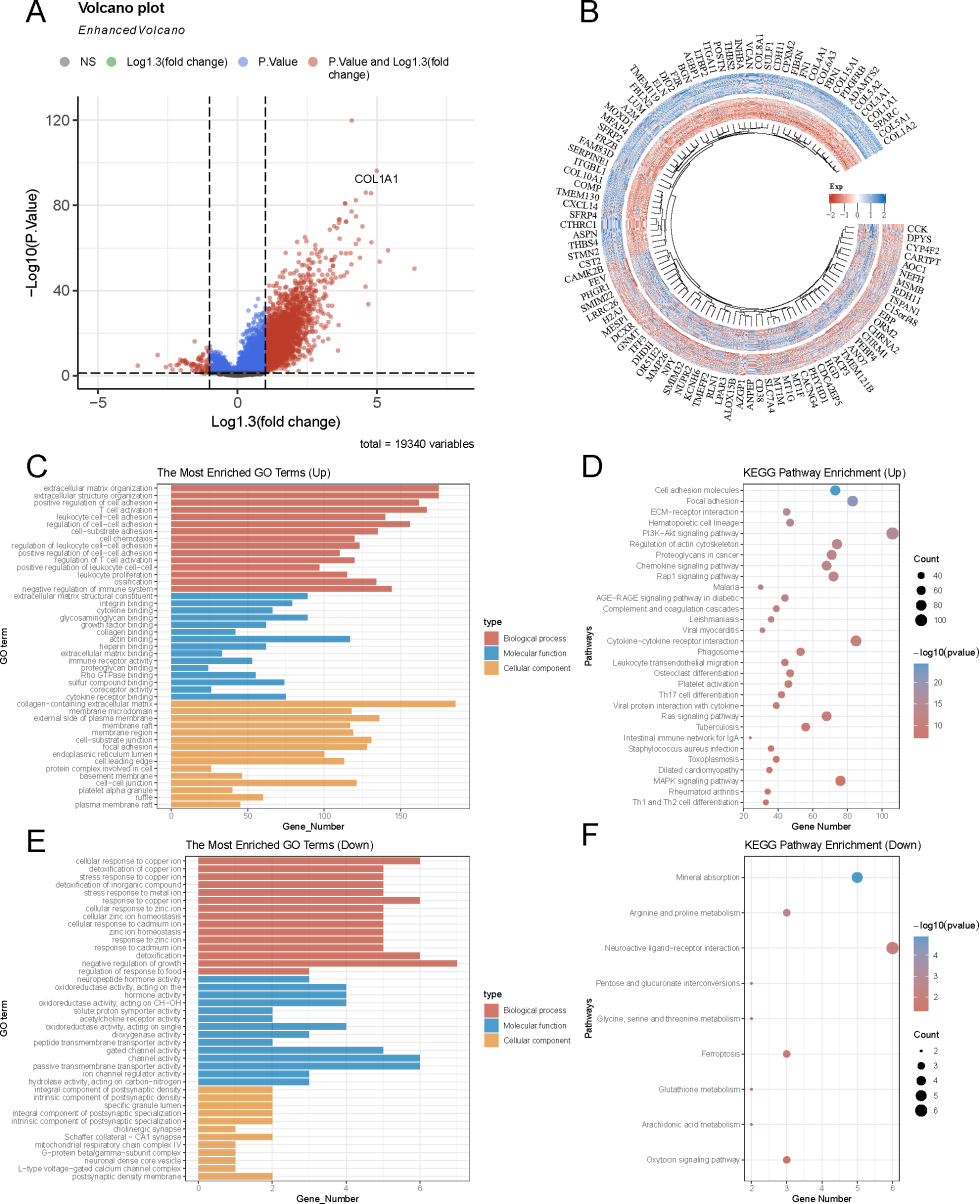
Identification and functional enrichment of ADAMTS2-coexpressed genes in PCa. **(A, B)** Scatter plots showing genes significantly positively or negatively related to ADAMTS2 expression in the TCGA-PRAD cohort. **(C, D)** GO and KEGG analyses of ADAMTS2 positively correlated genes, and **(E, F)** negatively correlated genes.

To elucidate the potential biological functions and molecular mechanisms underlying ADAMTS2-driven PCa progression, we performed a coexpression analysis via transcriptomic data from the TCGA-PRAD cohort. Genes exhibiting significant positive or negative correlation with ADAMTS2 expression were identified (**Figures 4A, B**). GO and KEGG pathway enrichment analyses illustrated that genes positively linked to ADAMTS2 were predominantly enriched in biological processes and pathways associated with the collagen-containing extracellular matrix, cytokine–cytokine receptor interaction, and the PI3K/AKT pathway (**Figures 4C, D**). In contrast, negatively correlated genes were primarily included in the negative control of cell growth and neuroactive ligand–receptor interaction (**Figures 4E, F**). These outcomes illustrate that ADAMTS2 may promote PCa progression by modulating extracellular matrix remodeling, inflammatory signaling, and key oncogenic pathways such as PI3K/AKT, thereby offering a molecular framework for its functional roles observed *in vitro*.

### ADAMTS2 stimulates the FAK/PI3K/AKT pathway in PCa cells

**Figure 5:**
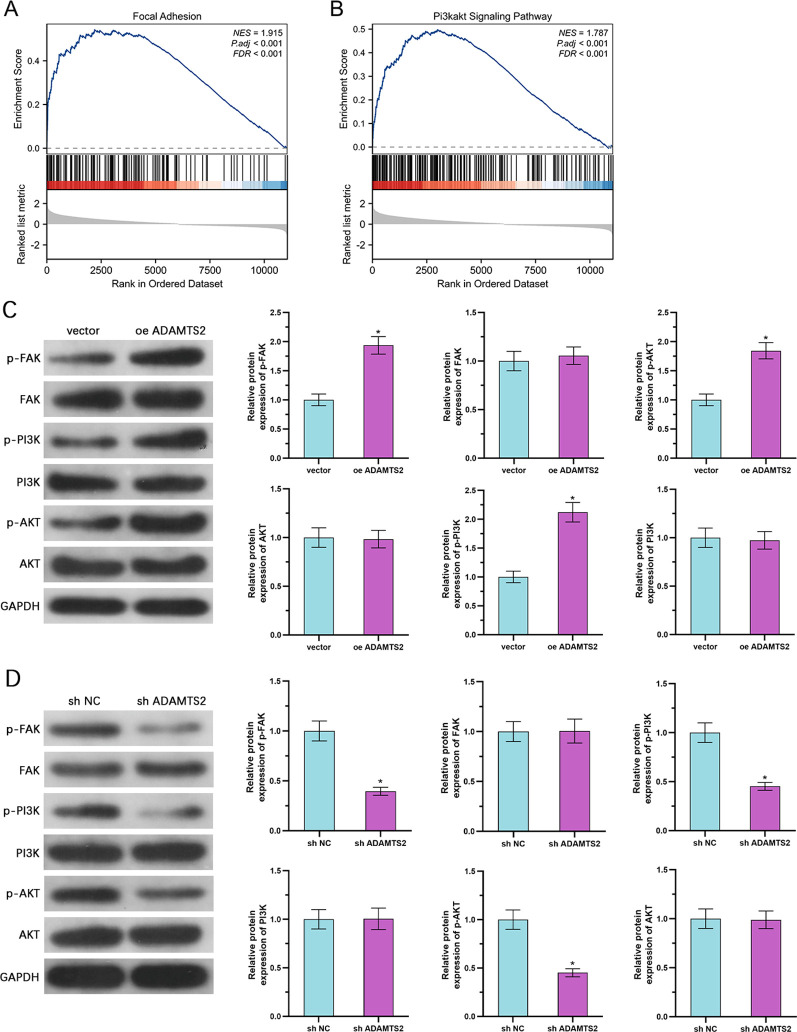
ADAMTS2 stimulation of the FAK/PI3K/AKT pathway in PCa cells. **(A, B)** GSEA of TCGA-PRAD data showing enrichment of PI3K/AKT signaling and focal adhesion gene signatures in tumors with high ADAMTS2 expression. **(C, D)** WB analysis evaluating the expression levels of phosphorylated (p-) and total FAK, PI3K, and AKT in **(C)** control vs. ADAMTS2-overexpressing DU145 cells, and **(D)** control vs. ADAMTS2-knockdown DU145 cells. GAPDH was used as the internal loading control. Representative immunoblots from three independent experiments are shown. Statistical significance was evaluated using an unpaired two-tailed Student’s t-test. *P < 0.05.

GSEA of the TCGA-PRAD dataset illustrated that ADAMTS2 overexpression was significantly related to enrichment of gene signatures linked to the PI3K/AKT pathway and focal adhesion (**Figures 5A, B**). Given the highly consistent functional phenotypes observed across multiple PCa cell lines in our initial assays, we selected DU145 cells as the primary representative model for subsequent in-depth mechanistic investigations. This decision was based on preliminary observations that DU145 cells exhibited superior experimental robustness, optimal transfection efficiency, and stable baseline tolerance to pharmacological probes compared to 22Rv1 cells, thereby minimizing data redundancy and ensuring highly reproducible mechanistic readouts. To validate this signaling link experimentally, we examined the phosphorylation status of key elements in the FAK/PI3K/AKT cascade in DU145 cells following ADAMTS2 modulation. WB showed that overexpression of ADAMTS2 notably elevated the phosphorylated FAK (p-FAK), PI3K (p-PI3K), and AKT (p-AKT) levels, while total protein levels of FAK, PI3K, and AKT continued to be unaltered (**Figure 5C**). Conversely, suppression of ADAMTS2 caused a pronounced decrease in the FAK, PI3K, and AKT phosphorylation without influencing their total expression (**Figure 5D**). These data demonstrate that ADAMTS2 promotes the stimulation of the FAK/PI3K/AKT axis in PCa cells, providing a mechanistic basis for its pro-tumorigenic effects.

### ADAMTS2 is coexpressed and physically interacts with COL1A1 in PCa

**Figure 6:**
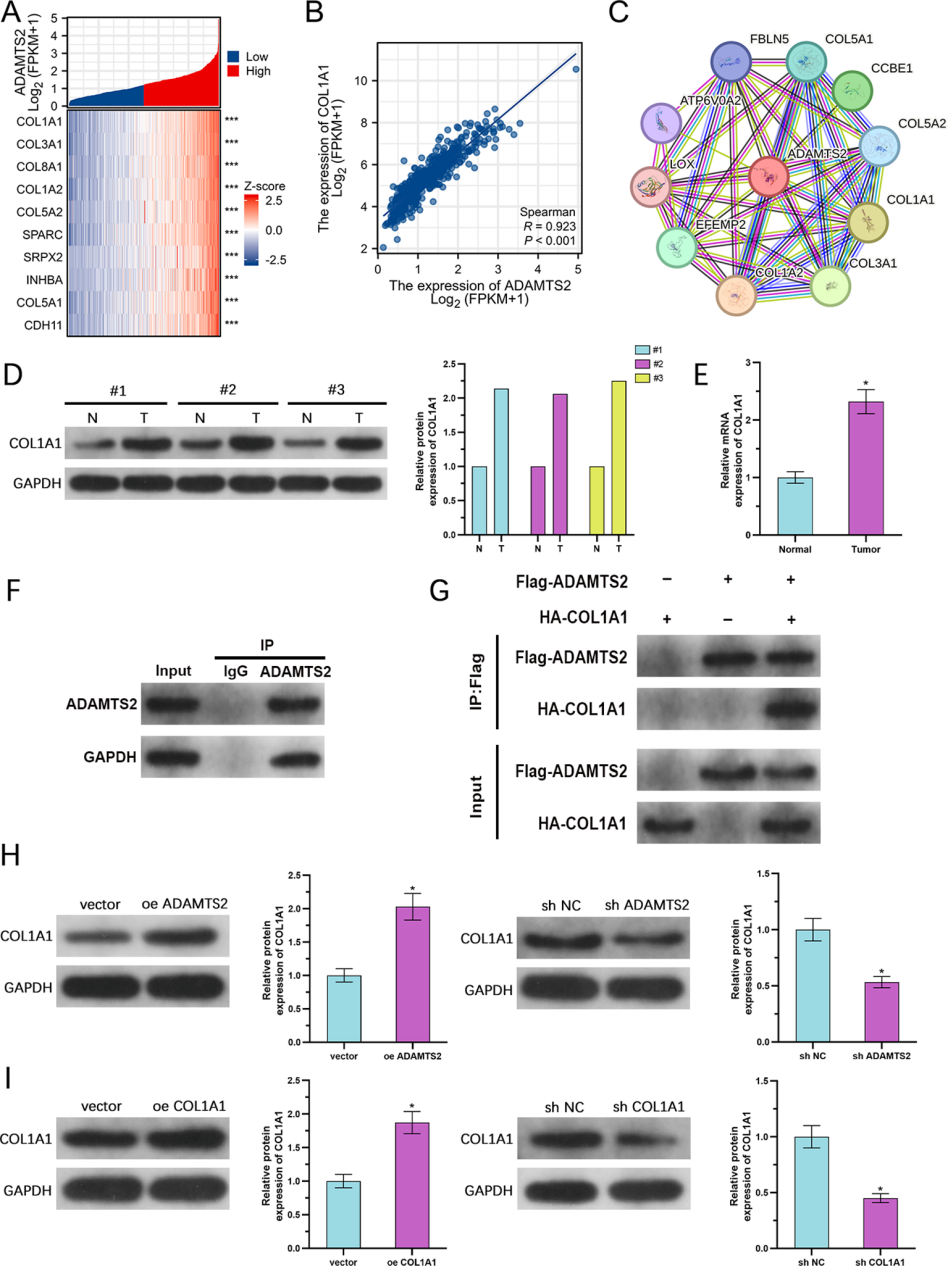
ADAMTS2 is coexpressed and physically interacts with COL1A1 in PCa. **(A)** Heatmap of the top 10 genes most strongly related to ADAMTS2 expression in the TCGA-PRAD cohort. **(B)** Scatter plot showing the correlation between ADAMTS2 and COL1A1 mRNA levels. **(C)** PPI network prediction from the STRING database indicating a potential interaction between ADAMTS2 and COL1A1. **(D, E)** Validation of COL1A1 mRNA (*n*=6 pairs) and protein (*n*=3 pairs) levels in paired human PCa and adjacent non-tumoral tissues using qRT-PCR and WB (paired two-tailed Student’s t-test). **(F, G)** Reciprocal Co-IP assessments illustrating physical interaction between endogenous and exogenously expressed ADAMTS2 and COL1A1 in DU145 cells. **(H)** WB of COL1A1 protein levels upon ADAMTS2 overexpression or knockdown in DU145 cells. **(I)** Evaluation of COL1A1 upregulation and downregulation effectiveness by WB in DU145 cells. Representative images from three independent experiments are shown. All quantitative data are presented as the mean ± SD from three independent biological replicates (n=3; unpaired two-tailed Student’s t-test). *P < 0.05, ***P < 0.001.

To evaluate the molecular partners of ADAMTS2 in PCa, coexpression analysis was conducted via the TCGA-PRAD transcriptomic dataset. Hierarchical clustering and heatmap visualization observed the top 10 genes most strongly correlated with ADAMTS2 expression (**Figure 6A**). Among these, COL1A1 exhibited the highest positive relationship (Spearman’s R = 0.923, P < 0.001), illustrating a potential functional relationship (**Figure 6B**). PPI network analysis via the STRING further predicted a direct or indirect interaction between ADAMTS2 and COL1A1 (**Figure 6C**). In line with these bioinformatic findings, WB and qRT-PCR analyses of paired clinical PCa specimens confirmed that COL1A1 protein and mRNA levels were significantly elevated in tumor tissues compared to neighboring adjacent non tumoral tissues (**Figures 6D, E**). To determine whether ADAMTS2 and COL1A1 physically associate, we performed Co-IP assays using both endogenous and exogenously expressed proteins in DU145 cells. Reciprocal Co-IP assessments illustrated a direct physical interaction between ADAMTS2 and COL1A1 (**Figures 6F, G**). Furthermore, WB revealed that ADAMTS2 overexpression upregulated COL1A1 protein levels, while ADAMTS2 knockdown markedly reduced COL1A1 expression (**Figure 6H**). The effectiveness of COL1A1 upregulation and downregulation in DU145 cells was verified by WB (**Figure 6I**). Collectively, these data establish a robust coexpression pattern and direct molecular interaction between ADAMTS2 and COL1A1 in PCa, positioning COL1A1 as a key downstream effector of ADAMTS2.

### ADAMTS2 confers resistance to ferroptosis in PCa cells through COL1A1

**Figure 7:**
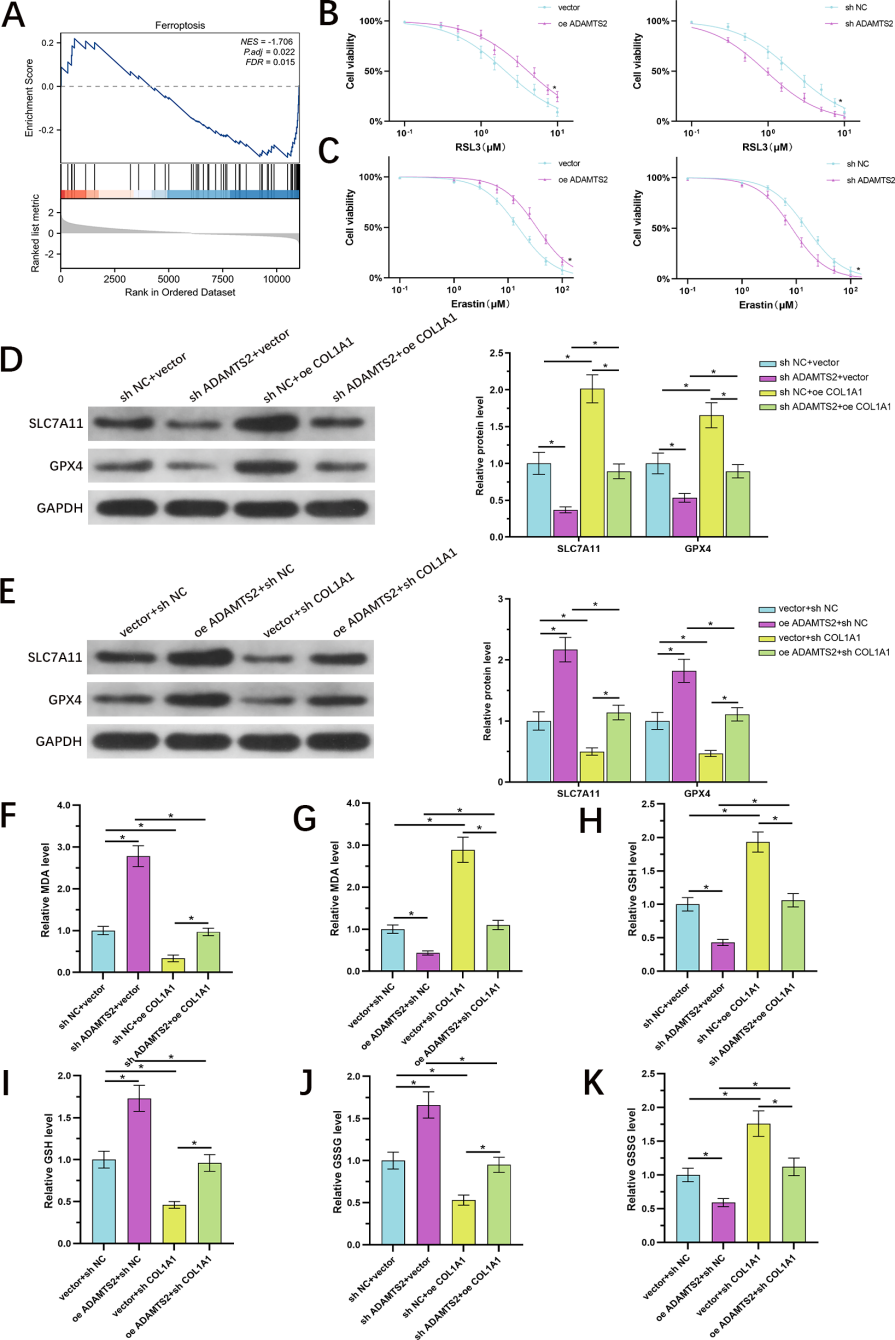
ADAMTS2 confers resistance to ferroptosis in PCa cells through COL1A1. **(A)** GSEA showing negative enrichment of ferroptosis-related gene signatures in TCGA-PRAD tumors with high ADAMTS2 expression. **(B, C)** Dose–response curves and IC_50_ values for ferroptosis inducers erastin and RSL3 in control, ADAMTS2-overexpressing, and ADAMTS2-knockdown DU145 cells. IC_50_ values were determined using non-linear regression analysis (n=3). **(D)** WB of SLC7A11 and GPX4 protein levels in ADAMTS2-knockdown cells in the presence or absence of COL1A1 overexpression. **(E)** WB of SLC7A11 and GPX4 in ADAMTS2-overexpressing cells in the presence or absence of COL1A1 knockdown. **(F, G)** MDA levels reflecting lipid peroxidation in the indicated cell groups. **(H–K)** Quantification of reduced GSH and oxidized GSSG under the specified genetic manipulations. All quantitative data are presented as the mean ± SD from three independent biological replicates (n=3). Statistical significance for multiple group comparisons was determined using one-way ANOVA followed by Tukey’s *post hoc* test. *P < 0.05.

Given the emerging function of ferroptosis in tumor suppression and the inverse relationship between ADAMTS2 expression and ferroptosis-correlated gene signatures observed in GSEA (**Figure 7A**), we hypothesized that ADAMTS2 may enhance PCa progression by suppressing ferroptosis. To assess this, we treated control and genetically modified DU145 cells with the ferroptosis inducers erastin and RSL3 and determined their half-maximal inhibitory concentrations (IC_50_). ADAMTS2 overexpression significantly increased the IC_50_ values for both erastin and RSL3, indicating reduced sensitivity to ferroptosis, whereas ADAMTS2 knockdown sensitized cells to these inducers (**Figures 7B, C**). At the molecular level, WB illustrated that ADAMTS2 suppression reduced the protein levels of main ferroptosis suppressors SLC7A11 and GPX4, and this reduction was rescued by ectopic overexpression of COL1A1 (**Figure 7D**). Conversely, ADAMTS2 overexpression upregulated SLC7A11 and GPX4, an effect that was attenuated upon COL1A1 silencing (**Figure 7E**). To assess functional consequences on lipid peroxidation—a hallmark of ferroptosis—we measured MDA levels. ADAMTS2 knockdown elevated MDA accumulation, which was reversed by COL1A1 overexpression (**Figure 7F**). In contrast, COL1A1 knockdown exacerbated MDA production in ADAMTS2-overexpressing cells (**Figure 7G**). Furthermore, we evaluated the cellular redox state by quantifying reduced GSH and oxidized GSSG. ADAMTS2 knockdown significantly decreased GSH levels and increased GSSG, indicative of oxidative stress; both effects were ameliorated by COL1A1 overexpression (**Figures 7H, J**). Conversely, COL1A1 silencing blunted the ADAMTS2-mediated increase in GSH (**Figure 7I**) and partially restored GSSG accumulation (**Figure 7K**). Together, these findings demonstrate that ADAMTS2 enhances ferroptosis resistance in PCa cells, and this protective action is critically dependent on its regulation of COL1A1 and subsequent modulation of the SLC7A11/GPX4 antioxidant axis and redox homeostasis.

### The oncogenic and anti-ferroptotic effects of ADAMTS2 are facilitated via the FAK/PI3K/AKT pathway

**Figure 8:**
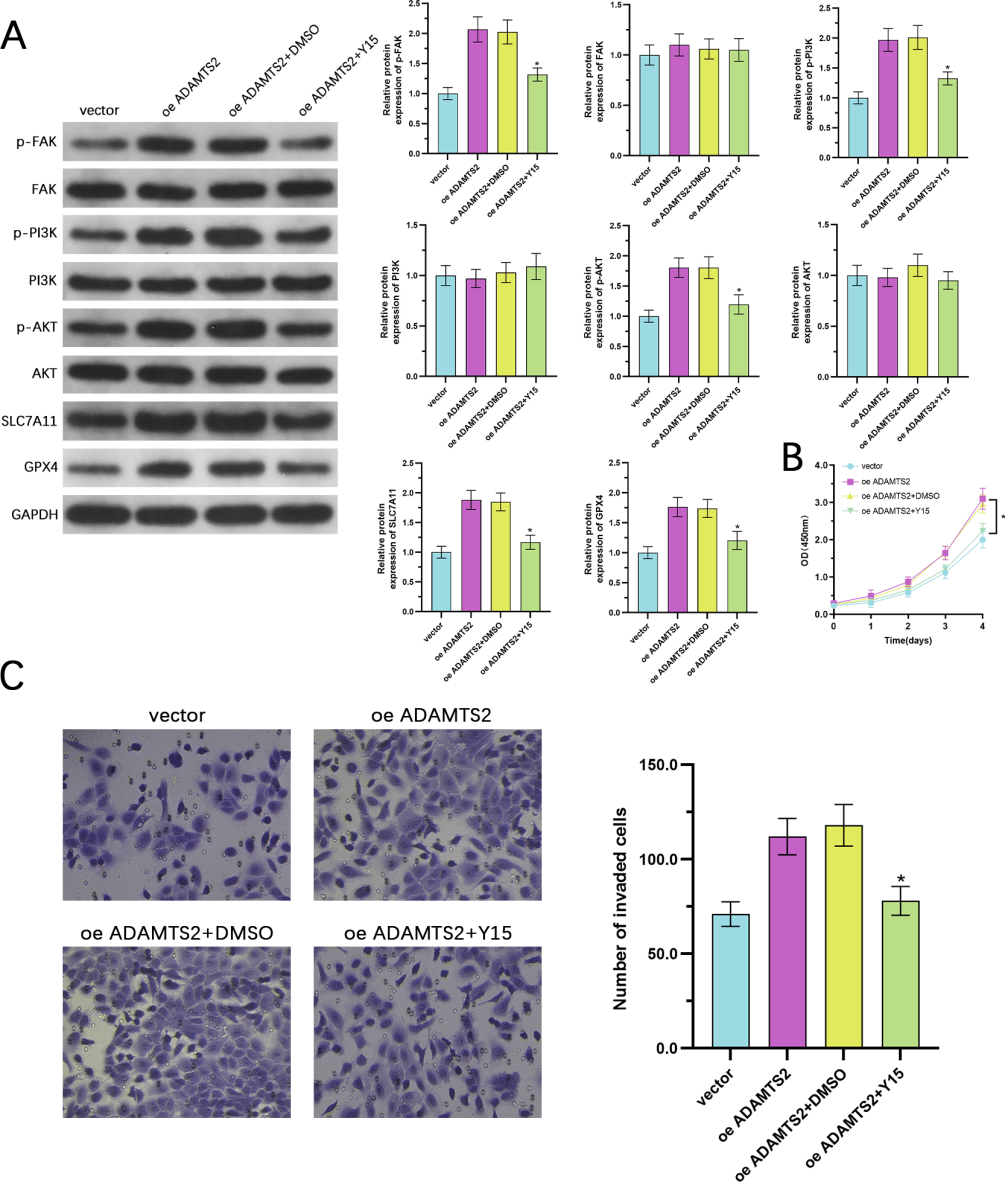
The oncogenic and anti-ferroptotic effects of ADAMTS2 are mediated via the FAK/PI3K/AKT pathway. **(A)** WB of p-FAK, p-PI3K, p-AKT, SLC7A11, and GPX4 in ADAMTS2-overexpressing DU145 cells treated with or without the FAK inhibitor Y15. **(B)** CCK-8 assay assessing cell proliferation in ADAMTS2-overexpressing DU145 cells with or without Y15 treatment. Data are analyzed using two-way ANOVA followed by Sidak’s multiple comparisons test. **(C)** Matrigel-coated Transwell invasion assay evaluating invasive capacity of ADAMTS2-overexpressing DU145 cells following Y15 administration. All bar graph quantitative data are presented as the mean ± SD from three independent biological replicates (n=3). Statistical significance for multiple group comparisons at a single time point was determined using one-way ANOVA followed by Tukey’s *post hoc* test. *P < 0.05.

To determine whether the tumor-promoting and ferroptosis-suppressive functions of ADAMTS2 depend on stimulation of the FAK/PI3K/AKT axis, ADAMTS2-upregulating DU145 cells were exposed to Y15, a selective inhibitor of FAK phosphorylation. WB showed that ADAMTS2 upregulation increased the p-FAK, p-PI3K, p-AKT levels, as well as the ferroptosis resistance markers SLC7A11 and GPX4; all these effects were markedly attenuated upon Y15 treatment (**Figure 8A**). Functionally, Y15 significantly reversed the enhanced proliferative capacity conferred by ADAMTS2 overexpression, as demonstrated by CCK-8 assays (**Figure 8B**). Likewise, Transwell invasion assays illustrated that Y15 abrogated the increased invasive potential of ADAMTS2-overexpressing DU145 cells (**Figure 8C**). These findings indicate that pharmacological inhibition of FAK signaling effectively counteracts both the pro-tumorigenic and anti-ferroptotic phenotypes induced by ADAMTS2, providing direct evidence that the FAK/PI3K/AKT pathway is a vital downstream effector mediating ADAMTS2’s oncogenic functions in PCa.

### Knockdown of ADAMTS2 suppresses tumor growth *in vivo* and downregulates FAK/PI3K/AKT signaling and ferroptosis resistance markers

**Figure 9:**
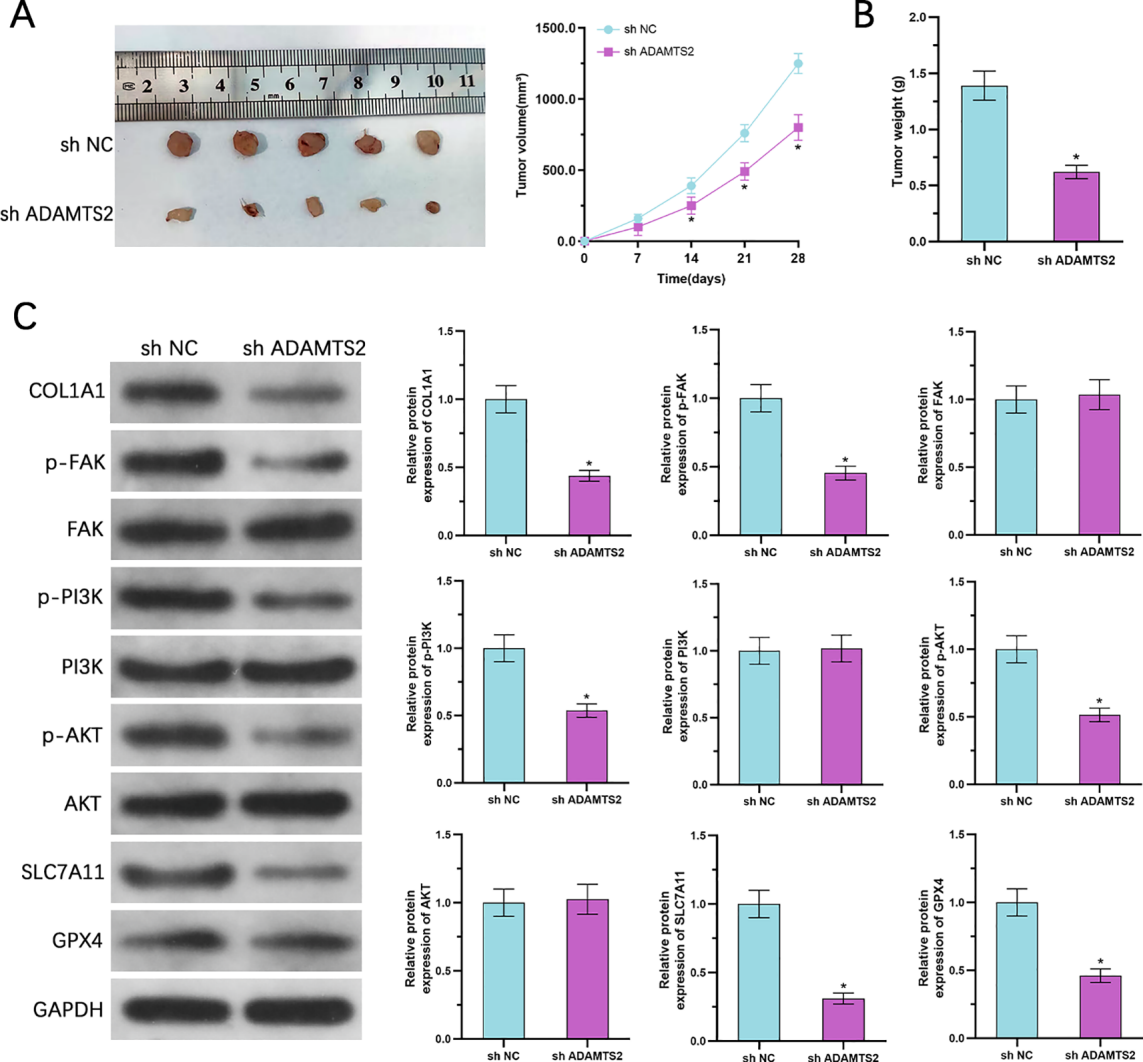
Suppression of ADAMTS2 suppresses tumor growth *in vivo* and downregulates FAK/PI3K/AKT signaling and ferroptosis resistance markers. **(A)** Tumor growth curves of xenografts derived from control or ADAMTS2-knockdown DU145 cells in nude mice, evaluated every 7 days over 28 days. (n=5 mice per group). Statistical significance was determined using two-way ANOVA followed by Sidak’s multiple comparisons test. **(B)** Representative photographs and quantification of tumor weight at the experimental endpoint. Data are presented as the mean ± SD (n=5 mice per group; two-tailed Student’s t-test). **(C)** WB of ADAMTS2, p-FAK, FAK, p-PI3K, PI3K, COL1A1, p-AKT, AKT, SLC7A11, and GPX4 in excised xenograft tumor tissues. Representative blots from three independent tumor samples per group are shown. All quantitative data are presented as the mean ± SD. *P < 0.05.

To assess the oncogenic function of ADAMTS2 in a physiological context, a xenograft tumor model was created by subcutaneously injecting nude mice with either control or ADAMTS2-knockdown DU145 cells. Tumor volumes were monitored every 7 days over a 28-day period. ADAMTS2 depletion resulted in significantly slower tumor growth than in the control group (**Figure 9A**). At the endpoint, excising and weighing the tumors were conducted, revealing a markable decrease in tumor volume and weight in the ADAMTS2-suppression cohort (**Figure 9B**). WB of the harvested tumor tissues demonstrated that ADAMTS2 knockdown led to decreased protein levels of COL1A1 and reduced phosphorylation of FAK, PI3K, and AKT. Concomitantly, expression of the ferroptosis defense proteins was substantially diminished (**Figure 9C**). These *in vivo* outcomes recapitulate our *in vitro* observations and confirm that ADAMTS2 promotes prostate tumor growth through stimulation of the FAK/PI3K/AKT pathway and inhibition of ferroptosis, thereby validating its role as a critical driver of PCa progression in a living organism.

## Discussion

PCa is a hormonally driven malignancy originating in the prostate gland, predominantly affecting older men and exhibiting a wide spectrum of biological behaviors—from latent, clinically insignificant lesions to highly aggressive, life-threatening disease (17). Its development is closely linked to androgen signaling, and while many cases progress slowly and may never require intervention, a subset evolves into advanced or metastatic forms that are resistant to standard therapies (18). The clinical management of PCa has improved with advances in imaging, biomarker development, and systemic treatments (19). However, the underlying biological determinants that dictate tumor behavior and response to therapy remain incompletely understood. Consequently, further investigation into the fundamental mechanisms governing PCa initiation and progression is imperative to refine prognostic models and develop more precise therapeutic strategies.

ADAMTS2 is a multidomain protein comprising a signal peptide, a prodomain, catalytic metalloproteinase and disintegrin-like domains, and multiple thrombospondin type-1 repeats, which collectively enable its substrate specificity and interaction with ECM components (20). Structurally, it requires calcium for stability and enzymatic activity, and its expression is primarily localized to fibroblasts and other stromal cells (21). Clinically, loss-of-function mutations in ADAMTS2 cause Ehlers-Danlos syndrome type VIIC, a rare connective tissue disorder marked by skin fragility and abnormal collagen architecture, underscoring its non-redundant role in collagen maturation (22). In oncology, ADAMTS2 exhibits dual and seemingly paradoxical functions: it constrains primary tumor growth by suppressing the formation of an immunosuppressive microenvironment, yet simultaneously promotes pulmonary metastasis—a dichotomy linked to its capability to control the innate immune landscape within the tumor microenvironment (23). Despite these advances in other cancers, no studies to date have examined ADAMTS2 expression patterns, regulation, or functional impact in PCa, highlighting a significant knowledge gap that this study aims to address. Here, we are the first to illustrate that ADAMTS2 is markedly upregulated in human PCa tissues at mRNA and protein levels, with elevated expression strongly correlated with aggressive clinicopathological features—including advanced T and N stage, higher Gleason score, residual tumor status, and older age—and significantly associated with shorter progression-free survival. Functionally, gain- and loss-of-function trials in multiple PCa cell lines reveal that ADAMTS2 potently enhances cell aggressiveness *in vitro*, while its stable knockdown robustly suppresses tumor growth in a xenograft mouse model. These outcomes verify ADAMTS2 as a novel oncogenic factor in PCa pathogenesis and a potential biomarker for disease aggressiveness and prognosis.

The collagen (COL) family constitutes the most abundant group of ECM proteins in mammals, presenting structural support and biochemical cues that control cell adhesion, migration, and signaling (24). Among its members, COL1A1 encodes the alpha-1 chain of type I collagen—the predominant fibrillar collagen in connective tissues such as bone, skin, and stroma (25). Type I collagen, a heterotrimer comprised two α1(I) and one α2(I) chains, is critical for tissue tensile strength and integrity (26). In cancer, COL1A1 is highly regarded as a structural scaffold and an active modulator of tumor behavior: elevated COL1A1 expression has been linked to desmoplasia, epithelial-mesenchymal transition (EMT), and poor prognosis in numerous cancers (e.g., breast, lung, and colorectal cancers) (27–29). In contrast, its role in PCa remains poorly characterized, with only limited evidence suggesting potential involvement in stromal remodeling and disease progression. Thus, a systematic investigation into COL1A1’s expression, regulation, and functional contribution in PCa is warranted. Our study addresses this gap by identifying COL1A1 as the top transcriptional correlate of ADAMTS2 in PCa through comprehensive coexpression analysis of the TCGA-PRAD cohort. We further illustrate that both mRNA and protein levels of COL1A1 are significantly higher in clinical prostate tumor specimens than in neighboring adjacent non tumoral tissues. Critically, we illustrate the first experimental evidence of a direct physical interaction between ADAMTS2 and COL1A1 in PCa cells via reciprocal co-immunoprecipitation assays. Moreover, we show that ADAMTS2 positively regulates COL1A1 expression at the protein level—knockdown of ADAMTS2 reduces COL1A1, while its overexpression enhances COL1A1 abundance. These findings position COL1A1 not only as a passive ECM component but as a key downstream effector of ADAMTS2, suggesting that the ADAMTS2–COL1A1 axis may drive PCa progression by reinforcing a tumor-permissive extracellular microenvironment that facilitates cell adhesion, migration, and pro-oncogenic signaling.

Ferroptosis, an iron-dependent type of controlled cell death, is driven by the overload of lethal lipid peroxides due to impaired antioxidant defenses, particularly the inactivation of GPX4 or dysfunction of the cystine/glutamate antiporter (system Xc^-^) (30, 31). Redox-active iron catalyzes Fenton reactions that promote peroxidation of polyunsaturated fatty acid-containing phospholipids, resulting in membrane impairment and cell death (32). Growing evidence illustrates that ferroptosis has a pivotal function in suppressing tumor growth and enhancing therapeutic efficacy in various cancers, including renal, liver, and breast carcinomas, thereby positioning it as an emerging target for cancer therapy (33, 34). In the context of PCa, our study reveals a previously unrecognized mechanism by which ADAMTS2 confers resistance to ferroptosis. We demonstrate that ADAMTS2 overexpression significantly blunts the cytotoxic effects of canonical ferroptosis inducers (erastin and RSL3), whereas its knockdown sensitizes cells to lipid peroxidation and oxidative stress. Mechanistically, this protective effect is mediated through the upregulation of main ferroptosis defense proteins—SLC7A11 and GPX4—along with enhanced glutathione synthesis and reduced malondialdehyde accumulation. Importantly, we establish that this anti-ferroptotic function is not autonomous but is tightly coupled to the FAK/PI3K/AKT signaling cascade: ADAMTS2 activates FAK phosphorylation, which in turn triggers downstream PI3K and AKT activation, and pharmacological inhibition of FAK with Y15 abolishes both the upregulation of SLC7A11/GPX4 and the ferroptosis resistance conferred by ADAMTS2. Given that AKT signaling has been shown to transcriptionally and post-translationally stabilize SLC7A11 and promote GPX4 activity, our findings position the ADAMTS2–FAK/PI3K/AKT axis as a critical upstream regulator of redox homeostasis in PCa cells. This mechanistic link not only expands the functional repertoire of ADAMTS2 beyond ECM remodeling but also implicates it as a potential barrier to ferroptosis-based therapeutic strategies in PCa.

Despite the compelling evidence presented herein, several limitations of our study warrant acknowledgment. First, our functional and mechanistic analyses were primarily conducted in established PCa cell lines, which may not fully recapitulate the heterogeneity, tumor microenvironment interactions, or androgen receptor signaling dynamics observed in primary human prostate tumors—particularly in treatment-naïve or castration-resistant contexts. Second, while we demonstrate a direct physical interaction between ADAMTS2 and COL1A1, the precise molecular interface and whether this interaction depends on ADAMTS2’s metalloproteinase activity remain to be elucidated. Third, our *in vivo* validation was limited to a subcutaneous xenograft model, which lacks the native prostate stromal architecture and immune components that could influence ADAMTS2 function *in situ*. Future studies employing genetically engineered mouse models (GEMMs) of PCa or patient-derived organoids would provide more physiologically relevant platforms to interrogate the ADAMTS2–COL1A1–FAK/PI3K/AKT–ferroptosis axis. Fourth, while our mechanistic investigation prioritized the FAK/PI3K/AKT cascade based on its robust quantitative enrichment and direct functional link to the ADAMTS2–COL1A1 interaction, our transcriptomic analyses also revealed the significant enrichment of other oncogenic networks, notably the MAPK signaling pathway. The specific contribution of MAPK signaling to ADAMTS2-driven aggressive phenotypes, and its potential compensatory crosstalk with the PI3K/AKT axis, warrants comprehensive exploration in future studies. Fifth, although we identify ADAMTS2 as a potential therapeutic target, specific pharmacological inhibitors are currently unavailable and have not yet been reported. Consequently, alternative strategies—such as the development of antisense oligonucleotides (ASOs), neutralizing antibodies targeting secreted ADAMTS2, or the pharmacological inhibition of its downstream FAK/PI3K/AKT signaling axis (as demonstrated with the FAK inhibitor Y15 in this study)—represent promising avenues for future clinical translation. Finally, although our data strongly establish a function for ADAMTS2 in promoting ferroptosis resistance, the potential crosstalk between this pathway and other forms of cell death or therapeutic modalities remains unexplored. Addressing these gaps will be necessary for the translation of ADAMTS2-targeted strategies into clinical applications.

## Conclusion

In brief, our investigation demonstrates that ADAMTS2 shows a significant upregulation in PCa and functions as an oncogenic driver by enhancing tumor cell aggressiveness. Mechanistically, ADAMTS2 interacts with and stabilizes COL1A1, resulting in stimulation of the FAK/PI3K/AKT pathway, which suppresses ferroptosis through upregulation of SLC7A11 and GPX4. These findings establish ADAMTS2 as a potential predictive biomarker and therapeutic target for PCa, particularly in strategies aimed at restoring ferroptosis sensitivity.

## Data Availability

The original contributions presented in the study are included in the article/**Supplementary Material**. Further inquiries can be directed to the corresponding authors.
