# Supplementary material for: ADAMTS2 drives prostate cancer progression by activating FAK/PI3K/AKT signaling and suppressing ferroptosis via COL1A1
Source: Front Oncol. 2026 Apr 27;16:1784882. doi: 10.3389/fonc.2026.1784882 (PMC13158085; doi:10.3389/fonc.2026.1784882)
Supplement: Supplementary file 1 [file DataSheet1.docx]

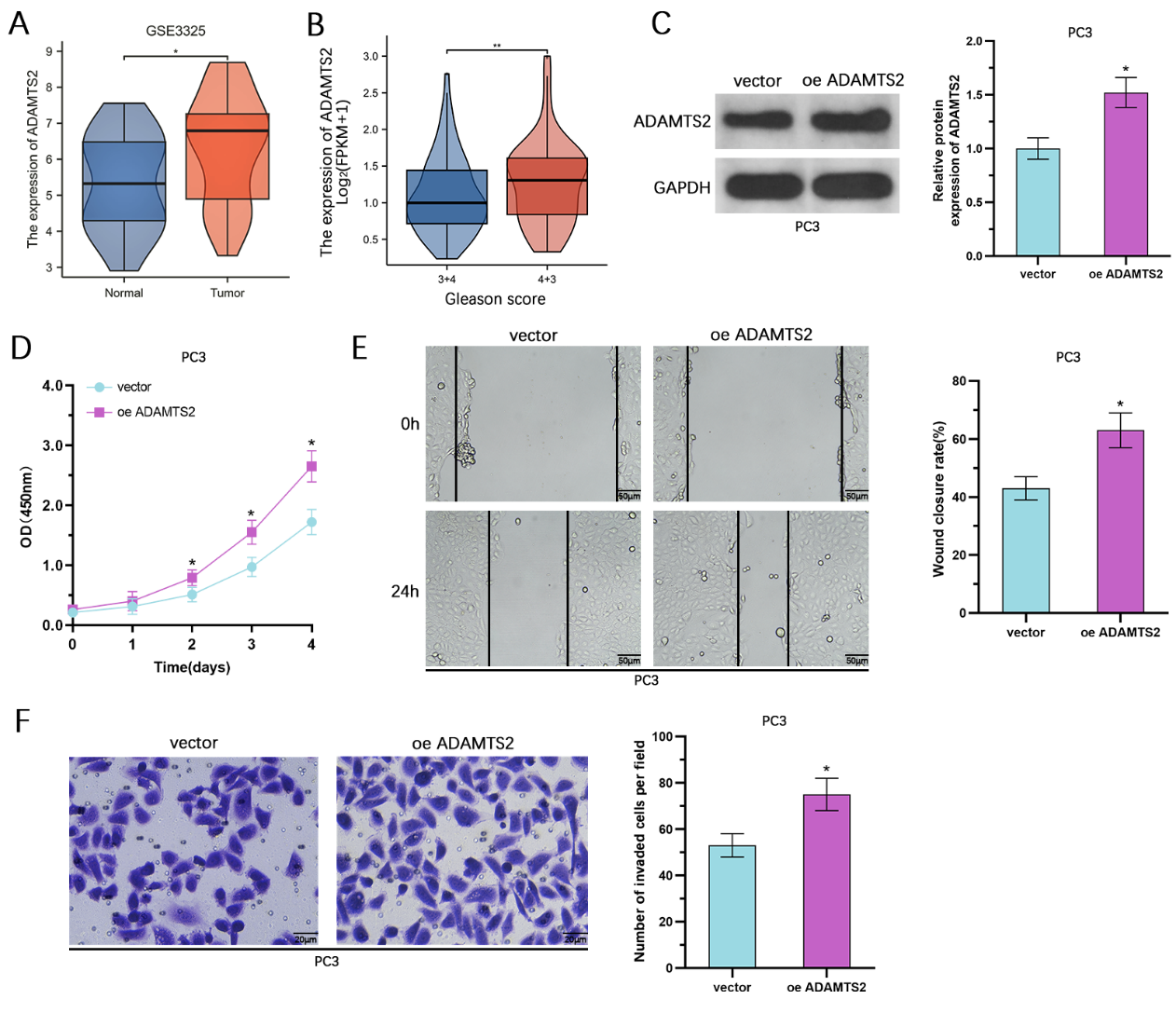


**Supplementary Figure 1. Clinical expression analysis of ADAMTS2 and validation of its biological functions in PC3 cells.**

(A) Analysis of ADAMTS2 mRNA expression levels in PCa tumor tissues and adjacent benign tissues using the GSE3325 dataset. (B) Stratification analysis of ADAMTS2 expression in PCa patients with a Gleason score of 7, comparing the 3+4 (ISUP Grade Group 2) and 4+3 (ISUP Grade Group 3) subgroups. (C) WB analysis of ADAMTS2 protein expression in PC3 cells following lentiviral transduction for overexpression. (D) CCK-8 assays evaluating the proliferation of control and ADAMTS2-overexpressing PC3 cells at 24, 48, 72, and 96 hours post-seeding. (E) Representative images and quantitative analysis of the wound healing assay assessing the migratory capacity of control and ADAMTS2-overexpressing PC3 cells. (F) Representative images and quantitative analysis of Matrigel-based Transwell assays evaluating the invasion ability of control and ADAMTS2-overexpressing PC3 cells. Data are shown as the mean ± SD from three independent biological replicates (n=3; two-tailed Student’s t-test). *P < 0.05, **P < 0.01.
